# Self-Locking Avoidance and Stiffness Compensation of a Three-Axis Micromachined Electrostatically Suspended Accelerometer

**DOI:** 10.3390/s16050711

**Published:** 2016-05-18

**Authors:** Yonggang Yin, Boqian Sun, Fengtian Han

**Affiliations:** Department of Precision Instrument, Tsinghua University, Beijing 100084, China; yinyg14@mails.tsinghua.edu.cn (Y.Y.); sbq12@mails.tsinghua.edu.cn (B.S.)

**Keywords:** MEMS accelerometer, three-component geophone, electrostatic suspension, cross-axis coupling, self-locking, nonlinear compensation, dynamic stiffness, seismic sensing

## Abstract

A micromachined electrostatically-suspended accelerometer (MESA) is a kind of three-axis inertial sensor based on fully-contactless electrostatic suspension of the proof mass (PM). It has the potential to offer broad bandwidth, high sensitivity, wide dynamic range and, thus, would be perfectly suited for land seismic acquisition. Previous experiments showed that it is hard to lift up the PM successfully during initial levitation as the mass needs to be levitated simultaneously in all six degrees of freedom (DoFs). By analyzing the coupling electrostatic forces and torques between three lateral axes, it is found there exists a self-locking zone due to the cross-axis coupling effect. To minimize the cross-axis coupling and solve the initial levitation problem, this paper proposes an effective control scheme by delaying the operation of one lateral actuator. The experimental result demonstrates that the PM can be levitated up with six-DoF suspension operation at any initial position. We also propose a feed-forward compensation approach to minimize the negative stiffness effect inherent in electrostatic suspension. The experiment results demonstrate that a more broadband linear amplitude-frequency response and higher suspension stiffness can be achieved, which is crucial to maintain high vector fidelity for potential use as a three-component MEMS geophone. The preliminary performance tests of the three-axis linear accelerometer were conducted under normal atmospheric pressure and room temperature. The main results and noise analysis are presented. It is shown that vacuum packaging of the MEMS sensor is essential to extend the bandwidth and lower the noise floor, especially for low-noise seismic data acquisition.

## 1. Introduction

Micromachined accelerometers are widely used in numerous areas, such as inertial navigation systems, smartphones, vehicle safety, tomography studies in oil and gas exploration, *etc.* [1,2,3,4]. In some situations, ultra-sensitive accelerometers are demanded to sense extremely weak motion or vibration information, such as the measurement of very weak acceleration in space missions [5,6] and land seismic wave acquisition [7,8,9]. An electrostatically-suspended accelerometer (ESA) usually employs a free proof mass (PM) levitated fully by capacitive position-sensing and electrostatic-forcing feedback, and can offer ultra-high resolutions better than pico-*g* in micro-gravity space applications by largely decreasing their measuring ranges below micro-*g* [6]. However, the traditional ESAs are mainly restricted in a few space applications [5,6] because of their large size, complicated fabrication techniques, and extremely high cost. Recently, several micromachined electrostatically-suspended accelerometers (MESAs) have been reported based on micro-electrical-mechanical system (MEMS) technologies [10,11,12,13,14,15,16,17,18]. MEMS-based fabrication can dramatically reduce the size and cost, and expand potential application possibilities. The operation principle of this servo-controlled accelerometer is based on the measurement of the electrostatic force necessary to maintain the PM motionless with respect to the sensor case. Since the PM has no mechanical support, many performance parameters of the MESA, such as range, sensitivity, stiffness, bandwidth, and resolution, can be readily tuned just by adjusting the electric parameters of the closed-loop suspension system [11,15]. Thus, the performance of a MESA device can be customized by different designs of the electrostatic suspension [18]. The PM of a MESA is typically suspended in the geometric center of the stator electrode cavity in six degrees of freedom (DoFs), which means the MESA has the ability to sense linear acceleration along three mutually-orthogonal axes [16,17,18]. Currently, the commercialized three-component (3C) geophones in land seismic sensing [19,20], such as DSU3 from Sercel (Carquefou Cedex, France) and SF3600 from Colibrys (Yverdon-les-Bains, Switzerland), utilize three single-axis accelerometers and position their sensitive axes in a mutually-orthogonal configuration, which will result in a bulky package. On the other hand, the three-axis MESA has the potential to offer broad bandwidth, high sensitivity, low noise and, thus, would be perfectly suited for use as a more compact 3C geophone.

Although the MESA has the potential to deliver high performance, relatively little work has been done to realize it, due to the difficult techniques required, such as complicated microfabrication, multiple-axis electrostatic suspension of the PM, and vacuum packaging. Among these techniques, one of the most challenging is position detection and levitation control of the PM in multiple DoFs. Currently, there are several reports on electrostatic suspension of the MESA with active control method, such as three-axis suspension of a spherical PM [10], five-axis levitation of ring-shaped PMs [11,12,13,14,16], and six-axis suspension of parallelepiped PMs [15,17,18]. However, considering inherent cross-axis coupling and the negative spring effect of electrostatic suspension in this three-axis MEMS accelerometer, how to lift up the PM smoothly from the resting electrodes to its equilibrium position during initial levitation, and how to eliminate the negative spring effect during normal suspension, are two of the most challenging aspects for designing a closed-loop electrostatic suspension system in practical MESA applications. It is expected that the PM can be levitated smoothly at any initial position and then suspended stably at its equilibrium position during normal device operation. So the electrostatic suspension of the MESA must function with two operating modes.

In the current work, we describe the electrostatic suspension design of a three-axis MESA employing a parallelepiped PM and discuss the experimental performance for potential use as a 3C MEMS geophone. During initial levitation where the PM is far away from its equilibrium position, the cross-axis coupling forces and torques between these axes are large as the PM is typically suspended by controllable electrostatic forces in multiple DoFs. In our previous experiments with a MESA suspended electrostatically in six DoFs [18], it was noticed that it is usually difficult to control, concurrently, the translation along the two in-plane axes (*x* and *y* axes) and the rotation around the out-of-plane axis (*z*-axis) effectively. In some situations, the PM stayed at its original position in these two DoFs even if their control voltages reached the allowable upper limit. A previous explanation of this phenomenon is that the lateral stop blocks may cause large static friction force, which is stronger than the maximum control force. By knocking the package of the MESA device to provide an extra impact force, then there is a probability to levitate the PM successfully in all six DoFs. In this work, by analyzing both the electrostatic force and torque in different attitudes of the PM before initial levitation, it is found that the PM may come into a self-locking zone when there exists an initial rotation angle around the *z*-axis. This gives us a clear explanation of this self-locking phenomenon and suggests an effective solution to the successful levitation of the mass. It is shown that the lateral coupling effect can be reduced greatly by setting a delayed operation for one in-plane axis, *i.e.*, the *x*-axis. With this control method, the experimental results have validated that the PM can be easily levitated up to its equilibrium position at any initial position, without need of knocking the case of the MESA device. On the other hand, stable suspension is crucial for normal device operation as the electrostatic suspension is inherent open-loop unstable [16]. One important consideration in the design of such closed-loop suspension systems is that the stiffness characteristics must match the full-scale range of the accelerometer. In this paper, an electrostatic suspension design is presented for the device operated at atmospheric pressure and in vacuum, respectively. A combination of feedback and a feed-forward compensation approach is proposed to eliminate the unstable negative spring effect and, thus, achieve more linear amplitude-frequency response and enhanced dynamic stiffness, which is important to achieve high vector fidelity in land seismic data acquisition. The preliminary performance tests of the three-axis accelerometer were conducted at atmospheric pressure and the main results are presented. It is shown that vacuum packaging of the MEMS device is essential to extend the bandwidth and lower the noise floor, especially for seismic sensing applications.

## 2. Design and Operation of the MESA

### 2.1. Device Structure

**Figure 1:**
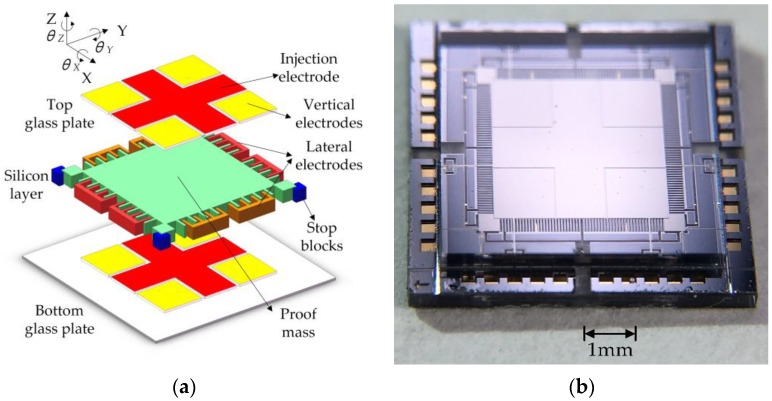
A glass/silicon/glass bonded structure of the MESA device: (**a**) three-dimensional exploded view; and (**b**) a close-up view of the fabricated device.

A typical structure of the MESA consists of two Pyrex 7740 glass plates and one silicon wafer, which is designed with a glass/silicon/glass triple-layer bonding structure and a compact electrode pattern as shown in Figure 1. The size of the fabricated device is 6.75 mm × 6.75 mm × 1.1 mm. The parallelepiped PM (mass: 2.76 mg, size: 3.5 mm × 3.5 mm × 88 μm) is surrounded by nine stator electrode pairs (one pair of injection electrodes, four pairs of vertical electrodes, and four pairs of lateral electrodes). The patterned vertical electrodes and injection electrodes are symmetrically arranged on the top and bottom glass plates. The free PM and the lateral electrodes are synchronously fabricated from the middle conductive silicon layer. Each lateral electrode has 28 comb teeth, which are designed to maximize sensing capacitances and, thus, sensitivity of the position sensors. A total of forty vertical stop blocks and eight lateral stop blocks are fabricated to restrict the PM’s range of motion and prevent possible contacting between the PM and the surrounding electrodes, as well as to effectively overcome the potential adhesion problem. The MESA device is fabricated with bulk micromachining based on the silicon on glass (SOG) technique [17,21]. To maintain stable suspension, the motion of the PM in all six DoFs, *i.e.*, the three translations along and the three rotations around the *x*, *y*, and *z* axes, is fully servo-controlled by capacitive position sensing and electrostatic actuation feedback through above electrode pairs.

### 2.2. Capacitive Position Sensing

**Figure 2:**
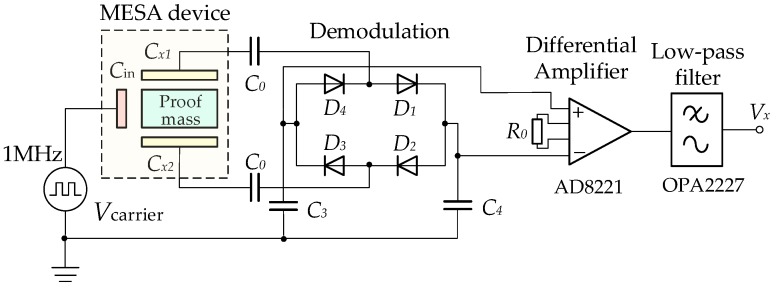
Schematic of the capacitive position sensing circuit.

A total of seven capacitive position sensing channels, from CH1 to CH7, are used to detect the motion of the PM in all six DoFs. The only difference among them is the gain setting of the amplifier in order to match different electrode capacitance ranges. In a single sensing channel, as schematically illustrated in Figure 2, a square-wave signal of amplitude 5 V and frequency 1 MHz is used as high-frequency carrier, which is capacitively-coupled to the PM through the common injection electrode capacitance, *C_in_*. Next, the difference between the electrode capacitance pair, *C_1_* and *C_2_* is converted into voltage signals and fed into a phase-sensitive demodulator based on a ring diode bridge. Finally, the position sensing signal is amplified with desirable sensitivity using a differential input-based instrument amplifier and low-pass filtered actively to provide the required bandwidth. The output, *V_x_*, is proportional to the differential capacitance variation and, thus, reflects the displacement of the PM.

Our previous MESA designs aim to produce an accelerometer with much smaller full scale range for potential space applications [17,18]. Therefore, the applied carrier amplitude is much smaller, ~140 mV, in order to minimize the negative spring effect stemming from the applied carrier voltage. By comparing with our previous designs, the full scale range of the *x* and *y* axes are increased greatly, ~0.3 g, for land seismic applications, and the *z*-axis range is designed at 3 g, where the unit “*g*” means gravitational acceleration. Therefore, the carrier amplitude is increased to 5 V and the preamplifier can be removed, which simplifies the capacitive sensing circuit and increases the signal-to-noise ratio.

### 2.3. Electrostatic Suspension

**Figure 3:**
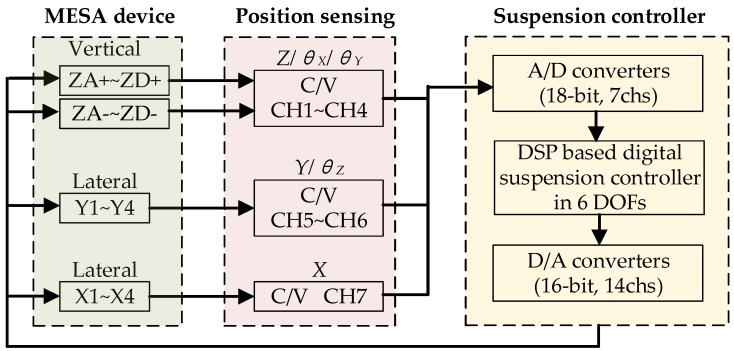
Schematic of the MESA suspension control system with six DoFs.

Unlike conventional MEMS accelerometers where a poof mass is physically attached to the substrate by a mechanical spring system, the MESA uses contactless electric support to suspend the PM. The operation of this force-balanced accelerometer is based on the measurement of the electrostatic force necessary to maintain the PM motionless with respect to the sensor housing. Figure 3 shows the basic operation principle of the three-axis accelerometer with electrostatic suspension in six DoFs. When an input acceleration is present, the PM displaces away from its nominal position and results in a change in differential capacitances between the movable mass and associated fixed electrodes. The position sensing circuit will detect these capacitance changes and output a set of position feedback signals. After A/D conversion, the seven position-sensing voltages will be used as feedback signals to a six-axis suspension control based on a digital signal processor (DSP) operated at a sampling frequency of 10 kHz. Note that an input transform matrix CL (7:6) and an output transform matrix LF (6:7) are realized in the DSP to match between the seven input/output channels and six single-axis suspension controllers [17]. Finally, seven pairs of differential control output signals are being D/A converted into 14 channel analog voltages and applied on the vertical and lateral fixed electrodes. Thus, the electrostatic feedback force will balance the input force and the PM will be maintained at its equilibrium position. In this closed-loop force balance operation, the control voltage is proportional to the input acceleration exerted on the PM [18].The scale factor of the accelerometer is designed as 30 V/g for the *x* and *y* axes and 2.5 V/g for the *z*-axis. For this sensor to be used as a geophone, the three-axis accelerometer will possess a relatively high bandwidth over 800 Hz after the device is packaged in vacuum.

## 3. Analysis and Prevention of the Self-Locking During Initial Levitation

### 3.1. Analysis of the Lateral Coupling Effect

**Figure 4:**
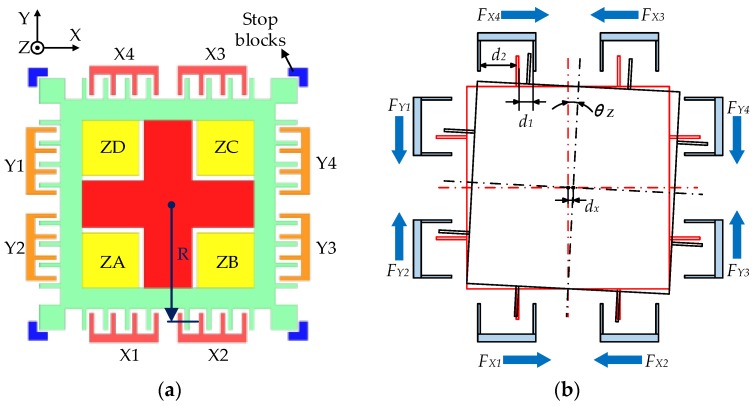
Top view of the electrodes pattern: (**a**) layout of the electrodes and stop blocks; and (**b**) the direction of the electrostatic force when the PM has a clockwise rotation around the *z*-axis and a translation along the *x*-axis.

Figure 4 shows the top view of the electrodes pattern. The gap between a movable comb tooth and its neighboring lateral electrodes is *d*_1_ or *d*_2_ (at null position: *d*_10_ = 6 μm, *d*_20_ = 29 μm). The nominal gap between the PM and a lateral stop block is 4 μm. To linearize the electrostatic force model, a DC preload voltage, 5 V in our design, is superimposed with the feedback control voltage and then applied in each electrode. If the PM has a clockwise rotation around the *z*-axis on the top view, the closed-loop suspension controller will generate a non-zero control voltage and applied on the *y*-axis electrodes, where the maximum control voltage is set at ±5 V. In our prior suspension design, the cross-coupling effect due to the forces generated by the *x*-axis electrodes was neglected since the rotation around the *z*-axis is only controlled by the *y*-axis electrodes. However, any non-zero rotation condition around the *z*-axis will change the balance of the *x*-axis electrostatic force and result in a cross-axis coupling torque around the *z*-axis rotation, even though the control voltage is set at 0 V and all electrode voltages are equal to the preload voltage. The electrostatic force between two parallel plates is given by:

(1) $$F_{e}=-\frac{\varepsilon A}{2}{\left(\frac{V_{e}}{d}\right)}^{2}$$

where *ε* is the permittivity of the air, *A* the effective plate area, *V_e_* the applied voltage, and *d* the distance, respectively. The minus sign means that the force is a kind of attractive force.

When there exists a small rotation angle θ_z_ (|θ_z_| ≤ 0.121° due to the stop blocks), the displacement of the lateral comb teeth along the *x*-axis direction can then be approximated by:

(2) $$x=R\cdot \text{sin}\theta_{Z}\approx R\cdot \theta_{Z}$$

where *R* is the average radius of the rotation (*R* = 1900 μm).

In addition, the translation along the *x*-axis (*d_x_*) will also change the gaps between the *x*-axis comb teeth and their neighboring electrodes. The range of *d_x_* is from −4 μm to 4 μm and the maximum of the rotation angle θ_z_ will decrease as *d_x_* increases.

The associated gaps (*d*_1_ and *d*_2_) of *x* and *y* axes are listed in Table 1. The applied electrode voltages (*V_e_*) in Equation (1) are listed in Table 2, where *V*_*X*1_~*V*_*X*4_ and *V*_*Y*1_~*V*_*Y*4_ represent the voltages on the associated electrodes. The solution case listed in Table 2 is just for comparison and will be introduced in Section 3.2.

For example, let *d*_x_ = 0, the electrostatic force generated by the X1 electrode is:

(3) $$F_{X1}=\frac{\varepsilon A}{2}\left({\left(\frac{V_{X1}}{d_{1}}\right)}^{2}-{\left(\frac{V_{X1}}{d_{2}}\right)}^{2}\right)$$

where *ε* = 8.85 × 10^−12^ F/m, *A* = 2.534 × 10^−8^ m^2^, *V_X1_* = 5.0 V, *d*_1_ and *d*_2_ are listed in Table 1.

The overall electrostatic torque around the *z*-axis direction is given by:

(4) $$T_{z}=(F_{X4}-F_{X3}+F_{X2}-F_{X1}+F_{Y2}-F_{Y1}+F_{Y4}-F_{Y3})\times R$$

**Figure 5:**
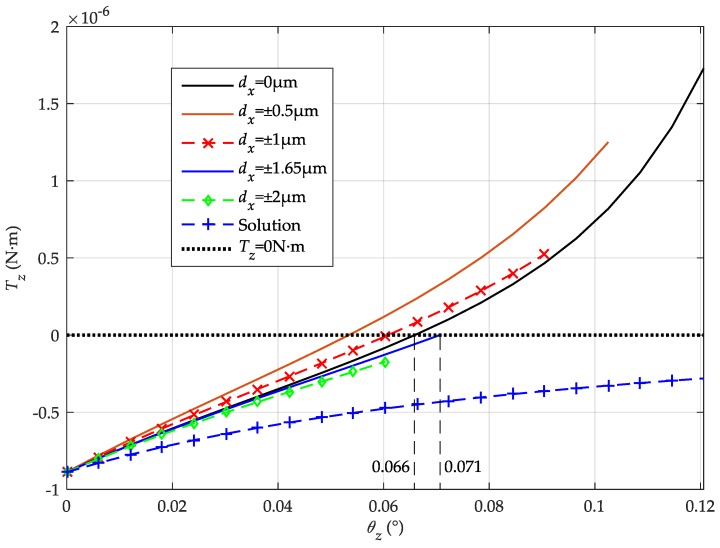
Electrostatic torque *vs.* initial rotation angle around the *z*-axis in different situations.

The calculated results of the combined torque over initial rotation angle are shown in Figure 5. If *T*_z_ > 0, the direction of the control torque is the same as the direction of the initial rotation, which means the PM will be pushed to the lateral stop blocks and be trapped in a self-locking zone.

Assuming *d*_x_ = 0, the critical angle of the self-locking zone is ±0.066° (55% of the entire angle range ±0.121°). When a small translation along the *x*-axis exists, the situation gets worse, e.g., the critical angle is further reduced to ±0.054° (53% of its angle range ±0.121°) for the case of *d*_x_ = ±0.5 μm. However, as |*d*_x_| increases, the probability of successful levitation will increase, too, because the critical angle will increase and the available rotation angle will shrink. For example, the critical angle is ±0.061° (60% of its angle range ±0.101°) when *d*_x_ = ±1 μm. If |*d_x_*| > 1.65 μm, the PM will not be trapped in the self-locking zone because its maximum rotation angle is limited below 0.071° and the electrostatic torque *T_Z_* is always negative. Thus the critical angle of the whole self-locking zone is 0.071° (59% of the entire angle range), which means if the rotation angle of the PM is above 0.071°, no matter what value *d*_x_ is, the PM will be trapped in the self-locking zone.

This result will explain the phenomenon that the PM can be levitated occasionally by just knocking its housing. The impact force generated by knocking may change the initial lateral position of the mass. If the PM leaves the self-locking zone, it will be controlled normally and levitated from its initial position to the geometry center of the electrode cavity. Otherwise, it will maintain at the self-locking condition and fail to be lifted up. To minimize the coupling effect on the *z*-axis rotation and eliminate the self-lock zone, we propose a solution aiming to delay the operation of the *x*-axis actuator until the *z*-axis rotation reaches its steady state.

### 3.2. A Solution to Prevent the Self-Locking Phenomenon

This paper provides a simple solution to solve the self-locking problem inherently existed in such MESA devices. According to the preceding analysis, the cross-axis coupling is the major source of the self-locking phenomenon. If the voltage on the *x*-axis electrodes is set at 0, as indicated in Table 2, when the rotation around the *z*-axis is being controlled during initial levitation, the cross-axis coupling between the *x*-axis translation and the *z*-axis rotation may not exist and the electrostatic torque is always negative, as shown in Figure 5. This delayed operation of the *x*-axis suspension control has been realized in a DSP-based suspension control program. The voltages of the *x*-axis electrodes are set to 0 until the other five DoFs (two translations along the *y* and *z* axes, three rotations around the *x*, *y*, and *z* axes) are levitated and maintained at their null positions. Using this method, the experimental results have validated that the PM can be levitated successfully in all six DoFs without the need of knocking its housing.

**Figure 6:**
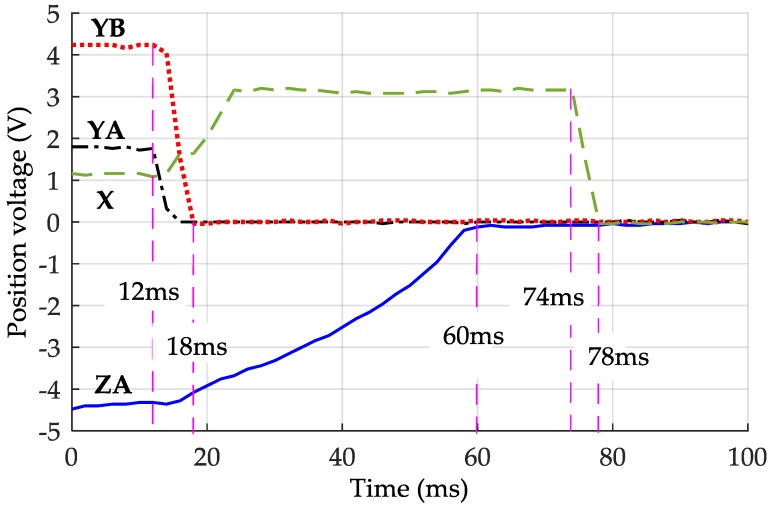
Measured time responses of four position sensing channels during initial lift-up of the PM.

Figure 6 shows time response of four position channels during initial levitation recorded by a digital storage oscilloscope. Upon applying the suspension control, the PM is levitated from its resting position to near the center of the electrode cavity. The solid line ZA represents the vertical position of the PM, which is detected by sensing the differential capacitance change of the electrode pair ZA (the electrode pairs ZB, ZC, and ZD are similar to ZA). The dot-dash line YA and the dotted line YB are the associated outputs of the differential electrode pairs Y1 and Y2, Y3, and Y4, respectively. The dashed line X represents the displacement of the *x*-axis (detected by four electrodes X1, X2, X3, and X4). Figure 6 shows that the levitation operation of the *z*-axis and the *y*-axis began at the moment *t* = 12 ms. The voltages of the YA and YB reached to near 0 at about 18 ms, while the voltage of the ZA reached 0 until *t* = 60 ms as the air-film damping effect along the vertical axes is much stronger than that along the lateral axes. During the time duration between 12 ms and 24 ms, it can be seen that the position voltage of the *x*-axis rose from 1.16 V to 3.12 V. This is because the *x*-axis motion was disturbed during initial levitation when the PM was moving along the *y*-axis and rotating around the *z*-axis. The only parameter which should be chosen in this solution is the moment when the *x*-axis control is activated. After the voltages of the YA, YB, and ZA all reached their steady states, *i.e.*, *t* > 60 ms, the suspension operation of the *x*-axis translation can be activated, at t = 74 ms in Figure 6. The entire lift-up operation of the PM is finally completed at *t* = 78 ms.

## 4. Electrostatic Suspension of the PM with Feed-Forward Compensation

### 4.1. Electrostatic Suspension System

Once the PM is levitated up and maintained at its equilibrium position as illustrated in Figure 6, the control system is switched to the normal suspension operation. As the suspension system will be working to keep the rotor centered in the electrode cavity with adequate suspension stiffness, it is assumed that only small displacement will occur in any direction. In this case, the complicated motion of the PM can be decoupled as six single-DoF suspension loops by neglecting small cross-coupling effects among the different DoFs. Here, we will take the *x*-axis suspension design as an example. The uncoupled equation governing the proof-mass dynamics in the *x*-direction is simply:

(5) $$m\ddot{x}=F_{x}-f\dot{x}+F_{d}$$

where *x* is the displacement of the PM from its nominal null position, *m* is the mass, *F_x_* is the electrostatic force acting on the mass, *f* is the damping coefficient, and *F_d_* is the external disturbances.

The electrostatic force can be linearized as [18]:

(6) $$F_{x}=K_{v}V_{x}+K_{x}x$$

where *K_v_* is the actuator gain, *V_x_* is the control voltage, and *K_x_* is the position stiffness.

Substituting Equation (6) into Equation (5) and then taking the Laplace transform to Equation (5) yields the transfer function governing the motion of the PM:

(7) $$G_{p}(s)=\frac{X(s)}{F_{d}(s)+K_{v}V_{x}(s)}=\frac{1}{ms^{2}+fs-K_{x}}$$

**Figure 7:**
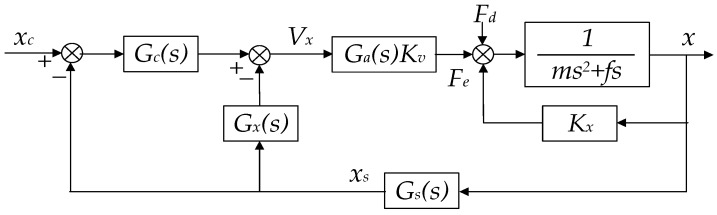
Block diagram of the electrostatic suspension system with feedforward compensation.

By inspecting the characteristic equation of the transfer function in Equation (7), open-loop instability due to the negative position stiffness, −*K_x_*, can be found as there is a pole on the right-hand side of the s-plane. Consequently, active feedback control must be employed to stabilize the suspension servo loop. A block diagram of the electrostatic suspension system is shown in Figure 7, where *G*_s_(s) is the transfer function of the position sensor, *G*_c_(s) is the linear feedback controller, *G*_x_(s) is the feed-forward compensator, and *G*_a_(s) is the transfer function of the drive amplifier. Both *G*_c_(s) and *G*_x_(s) will be designed in continuous-time domain and then realized digitally using the DSP.

The position sensing signal is low-pass filtered with a second-order Butterworth filter, which can be expressed mathematically as:

(8) $$G_{s}\left(s\right)=K_{s}\frac{w_{s}^{2}}{s^{2}+2\sqrt{2}w_{s}s+w_{s}^{2}}$$

where *K_s_* is the sensitivity of the sensing circuit and *w_s_* is the cutoff frequency of the low-pass filter.

For the MESA operated in an atmospheric environment, the air film between the movable PM and fixed electrodes can provide adequate damping. Thus, the feedback controller utilizes a typical lag compensator to stabilize the closed-loop system:

(9) $$G_{c}\left(s\right)=K_{c}\frac{1+T_{1}s}{1+T_{2}s}$$

where *K_c_* is the gain, and *T*_1_ and *T*_2_ are the time constant of the lag portion and *T*_2_ > *T*_1_. The lag compensation can greatly reduce the steady-state error of the compensated system.

The drive amplifier can be modeled as a first-order low-pass filter:

(10) $$G_{a}\left(s\right)=K_{a}\frac{1}{T_{a}s+1}$$

where *K_a_* is the voltage gain and *T_a_* is the time constant of the amplifier.

The model parameters and the lag compensators of the three-axis accelerometer operated at normal atmospheric pressure are listed in Table 3. Some model parameters (*K_v_* × *K_s_*, *f*, and *K_x_*) are obtained by fitting the experimental data with the theoretical model during the preliminary suspension test. The controller parameters are designed by considering the requirements, such as the suspension stiffness, stability margin, steady-state error, and dynamic response time.

### 4.2. Feed-Forward Compensation

Without the feed-forward compensator *G_x_*(s), the closed-loop transfer function of the electrostatic suspension system is:

(11) $$G_{cl}(s)=\frac{X_{s}(s)}{X_{c}(s)}=\frac{G_{k}(s)G_{c}(s)G_{p}(s)}{1+G_{k}(s)G_{c}(s)G_{p}(s)}=\frac{G_{k}(s)G_{c}(s)}{ms^{2}+fs-K_{x}+G_{k}(s)G_{c}(s)}$$

where $G_{k}(s)=K_{v}G_{s}(s)G_{a}(s)$.

**Figure 8:**
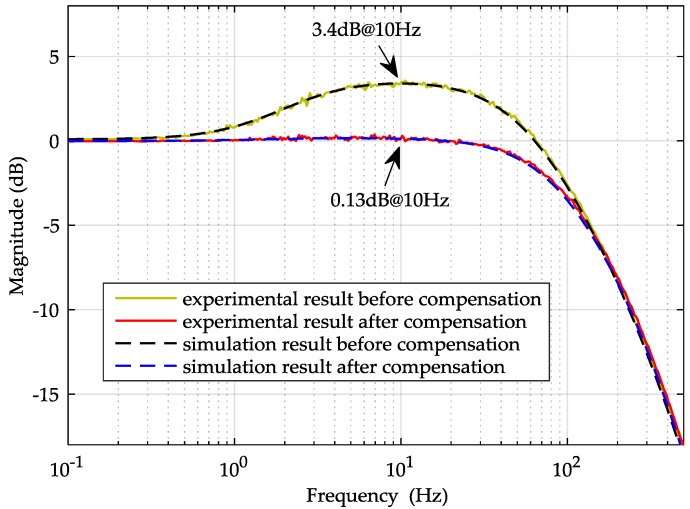
Simulated and experimental results of the x-axis closed-loop frequency responses.

The simulated closed-loop frequency response of Equation (11), using the model parameters listed in Table 3, is shown in Figure 8, along with the measured response using a dynamic signal analyzer, Agilent 35670 A (Agilent, Santa Clara, CA, USA). It is clearly seen that the experimental result is in good agreement with the simulated response. However, Figure 8 also indicates that the amplitude has been deviating from 0 dB gradually and reaches its peak 3.4 dB at 10 Hz, which will degrade the vector fidelity of recorded seismic wave data when the sensor is used as a high-resolution 3C geophone [19].

This kind of nonlinear amplitude-frequency characteristics is mainly caused by the negative position stiffness (*K_x_*) inherently in electrostatic suspension. The overall suspension stiffness of the closed-loop system, which represents the rejection ability to external disturbing force, is defined as [21]:

(12) $$K(s)=\frac{F_{d}(s)}{X(s)}=1/(\frac{X(s)}{F_{d}(s)})=1/(\frac{G_{p}(s)}{1+G_{k}(s)G_{c}(s)G_{p}(s)})=ms^{2}+fs-K_{x}+G_{k}(s)G_{c}(s)$$

**Figure 9:**
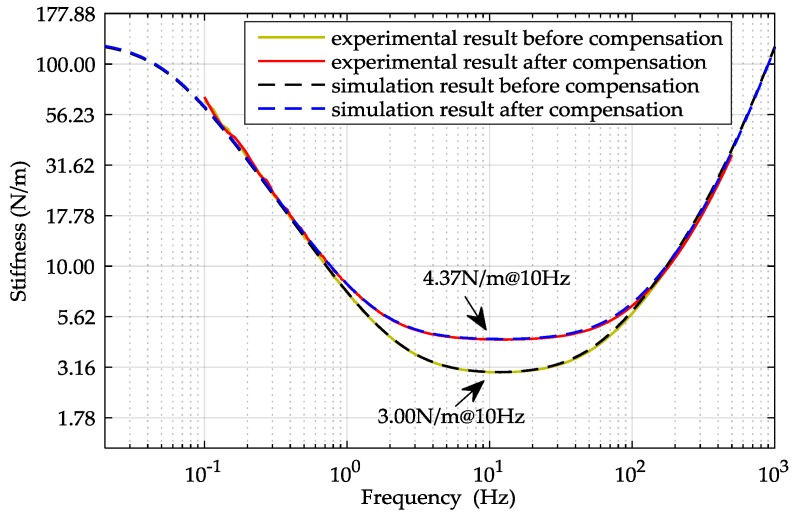
Simulated and experimental results of the *x*-axis suspension stiffness.

Equation (12) indicates that the existence of *K_x_* will decrease the overall suspension stiffness of the system. Both the simulation, using the model parameters listed in Table 3, and experimental result of the suspension stiffness before the feed-forward compensation are shown in Figure 9.

To acquire a more linear amplitude-frequency response, the overall system stiffness decrease caused by *K_x_* needs to be counteracted exactly. This is why the feed-forward compensator *G_x_*(s) is utilized in the closed-loop system [22].

Using the feed-forward compensation as illustrated in Figure 7, the suspension stiffness of the compensated system can be expressed as:

(13) $$K^{\prime }(s)=\frac{F_{d}(s)}{X(s)}=ms^{2}+fs+G_{k}(s)G_{c}(s)+(G_{k}(s)G_{x}(s)-K_{x})$$

If *G_x_*(s) is selected as:

(14) $$G_{x}(s)=\frac{K_{x}}{G_{k}(s)}=\frac{K_{x}}{K_{v}G_{s}(s)G_{a}(s)}$$

then, Equation (13) can be simplified as:

(15) $$K^{\prime }(s)=\frac{F_{d}(s)}{X(s)}=ms^{2}+fs+G_{k}(s)G_{c}(s)$$

By comparing Equation (15) with Equation (13), it is clear that the overall suspension stiffness is independent of *K_x_*. In this case, the closed-loop frequency response will be:

(16) $${G^{\prime }}_{cl}(s)=\frac{X_{s}(s)}{X_{c}(s)}=\frac{G_{k}(s)G_{c}(s)}{ms^{2}+fs+G_{k}(s)G_{c}(s)}$$

Considering that the position sensor and the drive amplifier have much higher bandwidth than the closed-loop system, over 14 kHz, as listed in Table 3, Equation (14) can be simplified as:

(17) $$G_{x}(s)\approx \frac{K_{x}}{K_{a}K_{v}K_{s}}$$

In our experiments, the *x*-axis feed-forward compensator was set to *G_x_*(s) = 1.7, which was calculated by Equation (17). The experimental results of the closed-loop frequency response and the suspension stiffness are shown in Figure 8 and Figure 9. Figure 9 shows that the suspension stiffness increases from 3.00 N/m to 4.37 N/m so that the negative stiffness (*K_x_* = 1.371 N/m) is exactly compensated by *G_x_*(s). Additionally, it is seen that a more broadband linear amplitude-frequency response can be achieved with the compensation in Figure 8. The results of the close-loop frequency response and the stiffness both agree well with the theoretical predication.

### 4.3. Simulation of the Suspension System in Vacuum

The MESA is composed of various noise sources, such as electronic noise, mechanical-thermal noise, and environment noise. If the MESA device is packaged in vacuum, the mechanical noise will be nearly removed. In addition, the bandwidth of the acceleration response can be extended dramatically because the air-film damping is so weak in a vacuum environment. To extend the bandwidth and lower the noise floor, vacuum packaging of the MEMS device is essential, especially for seismic sensing applications.

The controller design in a vacuum is a little different from that in air as the residual air-film damping can be negligible. Generally, the lead portion of the compensator is necessary to provide adequate force and achieve the required phase margin. To ensure the stable suspension in vacuum, a typical lead-lag compensator is utilized to illustrate the design procedure:

(18) $${G^{\prime }}_{c}\left(s\right)=K_{c}\frac{1+T_{1}s}{1+T_{2}s}\frac{1+t_{1}s}{1+t_{2}s}$$

where *K_c_* is the gain, and *T*_1_/*T*_2_ and *t*_1_/*t*_2_ are the time constants of the lag portion and lead portion, respectively. The lead portion can increase greatly the phase of the open-loop frequency response near the cutoff frequency, which will ensure desirable phase margin.

**Figure 10:**
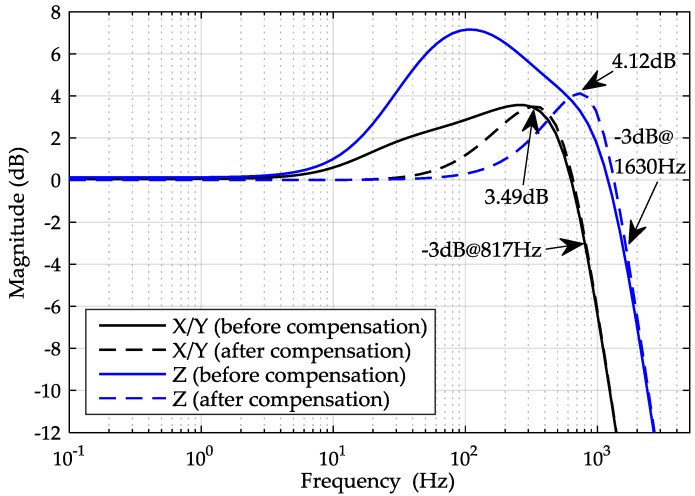
Simulated closed-loop frequency responses of the three-axis MESA in vacuum.

The principle of the feed-forward compensation in vacuum is similar to that in air. Applying the controller parameters in Table 4, a stable suspension system is achieved by simulation and the three-axis closed-loop frequency responses in vacuum condition are shown in Figure 10. The expected vacuum condition is below 10 Pa from initial evaluation on air-filming damping and Brownian noise. It is obvious from the simulation results that the closed-loop bandwidths are not very different for the compensated and uncompensated systems, whose bandwidth can all be increased above 800 Hz and is wide enough for use as a geophone. However, the large resonant peak of the uncompensated system in the intermediate frequency range illustrates clearly that this system has poor dynamic performance. It is clear that the feed-forward compensated system yields a more linear amplitude-frequency response over wide frequency range and improved dynamic performance.

## 5. Experimental Performance of the MESA

### 5.1. The MESA Setup

**Figure 11:**
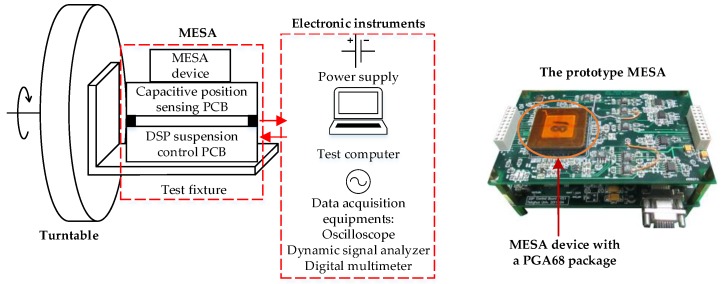
Experimental setup of the prototype MESA.

The experimental system that is used throughout this paper is a three-axis MEMS accelerometer with a thin parallelepiped PM supported electrostatically in all six DoFs. The schematic of the MESA setup is shown in Figure 11. The prototype MESA, which consists of a MESA device, a capacitive position sensing printed circuit board (PCB) stacked with the DSP-based suspension control PCB, was fixed on a precision turntable by the test fixture. The MESA device was atmospherically sealed in a PGA68 ceramic package and mounted on the sensing PCB, as shown in Figure 11. The digital control program was debugged using a computer and then uploaded to the DSP memory. Both the six-DoF feedback compensators and feed-forward compensators were run at a sampling frequency of 10 kHz. The accelerometer was tested by using the tilt method in the Earth’s gravitational field, where the input acceleration is a function of the angle between the sensitive axis and the local vertical. The input axis can be changed relative to the gravity vector by rotating the turntable to different positions.

### 5.2. Preliminary Experiment Results

The characteristics of the three-axis MEMS accelerometer have been tested for the device operated under normal atmospheric pressure and room temperature. The measured accelerometer performance for each input axis is summarized in Table 5 where the results for the *x*-axis are similar to those of the *y*-axis due to symmetrical configuration of the two lateral axes.

**Figure 12:**
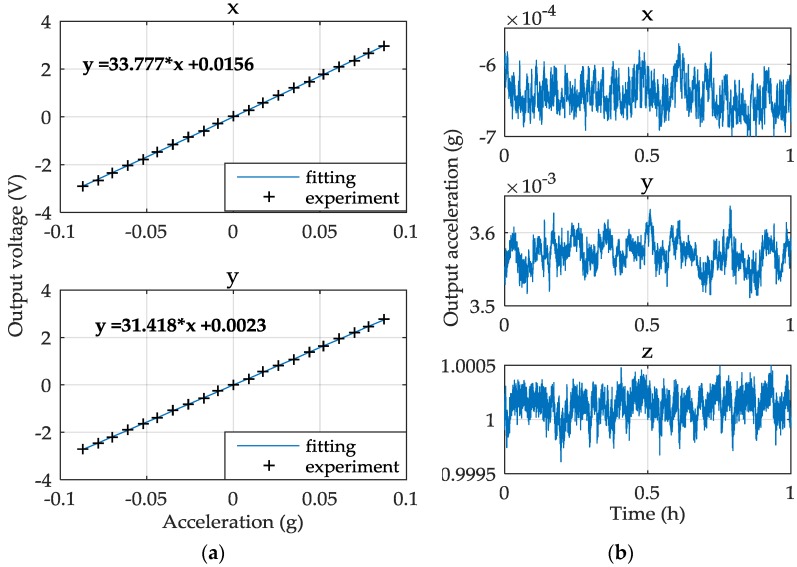
Experimental results: (**a**) the acceleration responses of the *x* and *y* axes; and (**b**) one-hour bias stability of the *x*, *y*, and *z* axes.

The MESA is calibrated in the Earth’s gravitational field where the input axis is changed relative to the gravity vector by rotating the table to different positions. The accuracy of the calibration is limited to less than 1.8 μg as the angle resolution of the turntable is 0.0001°. Figure 12a shows the acceleration responses of the *x* and *y* axes. The test angle ranges from −5° to +5° and the interval between two neighboring test points is 0.5°. The scale factor of the *x* and *y* axes is defined as the slope of the fitting line of the 21 test points. Considering that the *x*/*y*-axis measurements will be out of their ranges (~0.3 g) if the *z*-axis input is set to around 0 g, the measurement of the *z*-axis scale factor is a little different from the two lateral axes. In this work, we set the *z*-axis acceleration input to 1 g and −1 g and record the two output voltages, then the scale factor of the *z*-axis is defined as the slope of the two points. The nonlinearity is calculated as the percentage of the maximum offset voltage, which deviates farthest from the straight line among the 21 points, in the full scale output range, as shown in Figure 12a. In the resolution measurements, the accelerometer outputs are low-pass filtered at a bandwidth of 10 Hz and then recorded at a sampling frequency of 1 Hz via a 6.5 digit bench multimeter. Bias stability is one key performance for a high-performance accelerometer. After a thermal equilibrium process of 1 h, the bias stability test began. Figure 12b shows the 1-hour bias data of the *x*, *y*, and *z* axes. It should be indicated that the bias stability of the *z*-axis was tested under the gravity acceleration input (1 g).

### 5.3. Noise Performance and Analysis

The noise level of the MESA is evaluated by the power spectrum density (PSD), which can indicate the frequency characteristic of noise. The peak-to-peak noise PSD was measured using a dynamic signal analyzer (Agilent 35670 A), as listed in Table 5. It is clear that the noise floor has imposed heavy limitation on the MESA performance for this sensor use a 3C geophone. The measured noise of the MESA is mainly composed of thermo-mechanical noise, the position sensing noise, the digital controller noise, and the seismic noise in an Earth-bound test environment [23]. The thermo-mechanical noise (or Brownian noise) derives from the fact that the MEMS accelerometer consist of an electrostatically-suspended PM which is susceptible to the mechanical noise that results from molecular agitation. The contribution of Brownian noise on the *x*/*y* axes and the *z* axis is calculated to be 0.46 μg/Hz^1/2^ and 12.5 μg/Hz^1/2^, respectively. This difference is attributed to the air-film damping coefficient in the vertical motion of the mass being more than two orders of magnitude larger than that in the lateral motion. Vacuum packaging is an effective approach to minimize the air damping effect and Brownian noise can be negligible [24]. In addition to Brownian noise, the electronic noise in the position sensing electronics has contributed dominatingly to the noise floor of this accelerometer [25]. The contribution of the measured position sensing noise on the *x*/*y* axes and the *z* axis is calculated to be 3.4 μg/Hz^1/2^ and 32.3 μg/Hz^1/2^ in the signal bandwidth of 10 Hz, respectively. Additionally, the digital controller noise, which mainly arises from the A/D converters, analog multiplexer, and D/A converters, also worsen the noise floor. Therefore, the utilization of a capacitive sensing and suspension control ASIC with ultra-high signal-to-noise ratio (SNR) is preferable for ultra-low noise seismic sensing applications. Finally, the seismic noise level is a crucial problem in selecting the test site for such sensitive accelerometers. The experimental results indicate that isolation from external noise sources, such as seismic noise inherent in an Earth-bound test environment, is essential to improve the noise floor of the MESA [18]. The noise measurements should be performed in a quiet room with special ground mechanical decoupling.

## 6. Conclusions

The challenging self-locking problem during the MESA’s initial levitation is explained, and an effective solution is proposed and tested in this paper. If the initial rotation angle around the *z*-axis is over 59% of its allowable range, the cross-coupling force from the *x*-axis electrodes will be stronger than the maximum control force from the *y*-axis electrodes. In this case, the PM will be trapped in a self-locking region. We propose a simple solution by delaying the *x*-axis suspension operation realized in the DSP program, the PM can be levitated successfully in all six DoFs at any initial position with no need of knocking. The proposed method can prevent the self-locking issue effectively and will benefit the future engineering application of the MESA. It also offers a promising solution for initial levitation of such inertial sensors with multi-axis electrostatic suspension. Moreover, we propose a feed-forward compensation approach to minimize the negative stiffness effect inherently in electrostatic suspension. The experiment results demonstrate that more broadband linear amplitude-frequency response and higher suspension stiffness can be achieved, which is crucial to maintain superior vector fidelity for potential use as a compact three-component geophone. Finally, the preliminary performance tests of the three-axis accelerometer were conducted in normal atmospheric pressure and the main results are presented. To extend the bandwidth and lower the noise floor in seismic or vibration sensing applications, the completed device needs to be sealed in a vacuum package. Future work will focus on the noise performance improvement, which mainly includes optimization of the sensing and control electronics and realization of a chip-level vacuum packaging.

## Figures and Tables

**Table 1:** Lateral gaps between eight sets of comb tooth and their neighboring electrodes.

Electrode	*d_1_*	*d_2_*
X1	*d_10_* − *d_x_* + *x*	*d_20_* + *d_x_* − *x*
X2	*d_10_* + *d_x_* − *x*	*d_20_* − *d_x_* + *x*
X3	*d_10_* + *d_x_* + *x*	*d_20_* − *d_x_* − *x*
X4	*d_10_* − *d_x_* − *x*	*d_20_* + *d_x_* + *x*
Y1/Y3	*d_10_* + *x*	*d_20_* − *x*
Y2/Y4	*d_10_* − *x*	*d_20_* + *x*

**Table 2:** Applied voltages on eight lateral electrodes.

Case	*V_X1/X4_* (V)	*V_X2/X3_* (V)	*V_Y1/Y3_* (V)	*V_Y2/Y4_* (V)
*d_x_* = 0	5	5	10	0
*d_x_* > 0	0	10	10	0
*d_x_* < 0	10	0	10	0
**Solution**	**0**	**0**	10	0

**Table 3:** Model parameters of the MESA operated at atmospheric pressure.

Parameters	X/Y	Z
*K_v_* × *K_s_* (N/m)	X: 0.7954; Y: 1.300	75.48
*f* (Ns/m)	0.0095	7.0
*K_x_* (N/m)	1.371	16.88
*K_c_*	X: 165; Y: 105	180
*T_1_* (s)	0.1	0.2
*T_2_* (s)	3	6
*w_s_* (rad/s)	1.13 × 10^5^	1.13 × 10^5^
*K_a_*	1	1
*T_a_* (s)	1.10 × 10^−5^	1.10 × 10^−5^

**Table 4:** Deign parameters of the three-axis controllers in vacuum.

Parameters	X/Y	Z
*K_c_*	X: 320; Y: 204	16
*T*_1_ (s)	1/100	1/120
*T*_2_ (s)	1/2.5	1/3
*t*_1_ (s)	1/850	1/2200
*t*_2_ (s)	1/8500	1/30,000

**Table 5:** Summary of the experimental performance.

Performance	X	Y	Z
Range (g)	0.297	0.318	3.731
Scale factor (V/g)	33.777	31.418	2.681
Nonlinearity (%)	0.384	0.204	\
Resolution (μg)	34.9	26.2	\
Noise PSD (μg/Hz^1/2^)	9.3	10.1	54.6
1-h bias stability (μg)	13.2	17.8	111.4

## References

[1] Shaeffer D.K. (2013). MEMS inertial sensors: A tutorial overview. IEEE Commun. Mag..

[2] Zwahlen P., Dong Y., Nguyen A.M., Rudolf F., Stauffer J.M., Ullah P., Ragot V. Breakthrough in high performance inertial navigation grade Sigma-Delta MEMS accelerometer. Proceedings of the Position Location and Navigation Symposium (PLANS), IEEE/ION 2012.

[3] Mougenot D., Thorburn N. (2004). MEMS-based 3D accelerometers for land seismic acquisition: Is it time?. Lead. Edge.

[4] Qu H., Fang D., Xie H. (2008). A monolithic CMOS-MEMS 3-axis accelerometer with low-noise, low power dual-chopper amplifier. IEEE Sens. J..

[5] Rodrigues M., Foulon B., Liorzou F., Touboul P. (2003). Flight experience on CHAMP and GRACE with ultra-sensitive accelerometers and return for LISA. Class. Quantum Gravity.

[6] Touboul P., Metris M., Lebat V., Robert A. (2012). The MICROSCOPE experiment, ready for the in-orbit test of the equivalence principle. Class. Quantum Gravity.

[7] Mougenot D., Cherepovskiy A., Liu J. (2011). MEMS-based accelerometers: expectations and practical achievements. First Break.

[8] Jan M.T., Iqbal A., Shoaib M. An unconventional method for exploration of oil and gas. Proceedings of the 2012 International Conference on Emerging Technologies (ICET).

[9] Neeshpapa A., Antonov A., Agafonov V. (2015). A Low-Noise DC Seismic Accelerometer Based on a Combination of MET/MEMS Sensors. Sensors.

[10] Toda R., Takeda N., Murakoshi T., Nakamura S., Esashi M. Electrostatically levitated spherical 3-axis accelerometer. Proceedings of the IEEE 15th International Conference on Micro Electro Mechanical Systems.

[11] Houlihan R., Kraft M. (2002). Modeling of an accelerometer based on a levitated proof mass. J. Micromech. Microeng..

[12] Murakoshi T., Endo Y., Fukatsu K., Nakamura S., Esashi M. (2003). Electrostatically levitated ring-shaped rotational-gyro/accelerometer. Jpn. J. Appl. Phys..

[13] Nakamura S. MEMS inertial sensor toward higher accuracy and multi-axis sensing. Proceedings of the 4th IEEE Conference on Sensors.

[14] Kraft M., Damrongsak B. Micromachined gyroscopes based on a rotating mechanically unconstrained proof mass. Proceedings of the 9th IEEE Conference on Sensors.

[15] Cui F., Liu W., Chen W., Zhang W., Wu X. (2011). Design, fabrication and levitation experiments of a micromachined electrostatically suspended six-axis accelerometer. Sensors.

[16] Han F., Wang L., Wu Q., Liu Y. (2011). Performance of an active electric bearing for rotary micromotors. J. Micromech. Microeng..

[17] Ma G., Han F., You P., Yan X. (2015). Experimental study of a low-g micromachined electrostatically suspended accelerometer for space applications. Microsyst. Technol..

[18] Han F., Sun B., Li L., Wu Q. (2015). Performance of a sensitive micromachined accelerometer with an electrostatically suspended proof mass. IEEE Sens. J..

[19] Li G., Chen G., Zhong J. (2009). Analysis of geophone properties effect for land seismic data. Appl. Geophys..

[20] Laine J., Mougenot D. Benefits of MEMS based seismic accelerometers for oil exploration. Proceedings of the TRANSDUCERS & EUROSENSORS ’07.

[21] Zhang T., Zhou B., Yin P., Chen Z., Zhang R. (2016). Optimal Design of a Center Support Quadruple Mass Gyroscope. Sensors.

[22] Han F., Gao Z., Li D., Wang Y. (2005). Nonlinear compensation of active electrostatic bearings supporting a spherical rotor. Sens. Actuators A Phys..

[23] Li G., Wu S., Zhou Z., Bai Y., Hu M. (2013). Design and validation of a high-voltage levitation circuit for electrostatic acceleromters. Rev. Sci. Instrum..

[24] Merdassi A., Yang P., Chodavarapu V.P. (2015). A wafer level vacuum encapsulated capacitive accelerometer fabricated in an unmodified commercial MEMS process. Sensors.

[25] Josselin V., Touboul P., Kielbasa R. (1999). Capacitive detection scheme for space accelerometers applications. Sens. Actuators A Phys..

